# Synergistic impact of chrono-nutritional and pro-inflammatory dietary patterns on chronic pain recovery: a prospective cohort study with population-based corroboration

**DOI:** 10.3389/fnut.2026.1798285

**Published:** 2026-07-15

**Authors:** Chenxi Liu, Yiping Tong, Mingxia Pan, Xiang Chen, Xuan Yang, Fuhuai He, Xiangyang Cao, Jinjuan Tian, Keke Guo, Yang Li, Yanlei Wang, Linbo Xing

**Affiliations:** 1 Luoyang Orthopedic Hospital of Henan Province (Henan Provincial Orthopedic Hospital), Luoyang, China; 2 School of Nursing, Fujian University of Traditional Chinese Medicine, Fuzhou, China

**Keywords:** chronic pain, chrono-nutrition, Dietary Inflammatory Index, dietary patterns, Eating Jetlag, synergistic interaction, systemic inflammation

## Abstract

**Background:**

Current research often overlooks synergistic interactions within dietary patterns, specifically between dietary composition and chrono-nutrition. Using Lumbar Disc Herniation (LDH) as a model of chronic inflammatory pain, this study examined whether meal-timing misalignment, quantified as Eating Jetlag (EJL; the absolute difference in eating midpoint between workdays and freedays), and a pro-inflammatory diet, quantified by the Dietary Inflammatory Index (DII), were associated with poorer recovery.

**Methods:**

This two-stage study first employed a prospective longitudinal cohort of 324 LDH patients undergoing conservative treatment with 6-month follow-up. Baseline data on DII, EJL, and serum interleukin-6 (IL-6) were collected. Growth Mixture Modeling (GMM) identified functional recovery trajectories using ODI scores. Latent Profile Analysis (LPA), correlation/network analysis, and exploratory Structural Equation Modeling (SEM) were then used to characterize baseline diet-rhythm-inflammation features and their associations with symptoms. Second, weighted NHANES data from 2009–2010, 2015–2016, and 2017–2018 were analyzed as population-level corroboration.

**Results:**

GMM identified three recovery trajectories, with 23.8% of patients classified in the chronic persistent group. Compared with the Rapid Recovery group, the Chronic Persistent group had higher DII (0.85 ± 1.45 vs. 0.31 ± 1.41, *p* = 0.030), higher IL-6 (5.99 ± 1.06 vs. 5.56 ± 1.17, *p* = 0.021), worse sleep score (11.55 ± 3.35 vs. 7.22 ± 3.92, *p* < 0.001), and higher pain VAS (6.34 ± 1.96 vs. 1.00 ± 0.95, *p* < 0.001). Restricted cubic spline analysis showed a significant overall association between DII and poor recovery (*p* = 0.018), whereas the nonlinear component was not significant (*p* = 0.333). In multivariable analysis, sleep score, pain VAS, and baseline ODI remained independently associated with poor recovery. Exploratory SEM showed that DII was strongly associated with IL-6, EJL was associated with sleep disturbance, and pain VAS was strongly associated with baseline ODI; however, global model fit was suboptimal. In weighted NHANES analyses, higher DII remained associated with prevalent low back pain.

**Conclusion:**

Chrono-nutritional misalignment and pro-inflammatory diets were associated with less favorable LDH recovery. These findings support further investigation of diet-rhythm-inflammation features in LDH prognosis and suggest that immuno-metabolic and behavioral factors may be clinically relevant adjunctive targets.

## 1 Introduction

Low back pain (LBP) has become a leading cause of disability globally, imposing a substantial burden on healthcare systems and the socio-economy (1). As a common etiology of LBP (2), the primary management strategy for Lumbar Disc Herniation (LDH) favors conservative treatment, aiming to alleviate symptoms through intrinsic immunomodulation and tissue repair mechanisms (3, 4). However, clinical observations indicate significant individual heterogeneity in the prognosis of conservative care: while some patients achieve rapid recovery within weeks, a considerable proportion experience a protracted course that eventually evolves into refractory chronic low back pain. Traditional biomechanical models, such as the degree of disc herniation or the extent of spinal stenosis, often fail to adequately explain these disparities in clinical outcomes. This necessitates expanding our perspective from local anatomical structures to the organism’s systemic biological environment, particularly focusing on modifiable lifestyle factors like dietary patterns.

Current nutritional science often focuses on individual nutrients or isolated dietary components. However, this reductionist approach may overlook the complex interactions within an overall dietary pattern. In recent years, the emergence of immunometabolism theory has provided a novel perspective for understanding pain chronification. Accumulating evidence suggests that persistent low-grade systemic inflammation not only hinders the natural repair of damaged tissues but also drives central sensitization by activating glial cells and sensitizing peripheral nociceptors (5, 6). Diet, as one of the controllable environmental factors regulating systemic inflammation levels, has garnered increasing attention for its potential pro-inflammatory effects. The Dietary Inflammatory Index (DII) has been proven to be closely related to various chronic diseases, yet its specific role in the LBP recovery trajectory remains insufficiently elucidated (7).

However, a comprehensive dietary pattern encapsulates not only “what to eat (composition)” but also “when to eat (timing).” Emerging research in chrono-nutrition points out that the misalignment between feeding behavior and biological rhythms—specifically the variation in meal timing patterns between workdays and free days, termed “Eating Jetlag” (EJL) (8)—can lead to desynchronization between peripheral clocks (e.g., liver and gut) and the central clock. Mechanistically, this disruption of circadian rhythms can attenuate the anti-inflammatory effects of glucocorticoids and abnormally upregulate the expression of pro-inflammatory cytokines (such as IL-6 and TNF-*α*) (9), thereby rendering the organism in a susceptible pro-inflammatory state.

Based on this background, we hypothesized that higher DII and greater EJL would be associated with poorer 6-month functional recovery in LDH patients receiving conservative treatment, and that IL-6, sleep disturbance, and pain severity might characterize this association. To examine this hypothesis, we first analyzed a prospective clinical cohort and then used the National Health and Nutrition Examination Survey (NHANES) as a population-level corroborative dataset rather than a formal external validation dataset. This study aimed to provide biologically informed lifestyle clues for identifying patients at risk of delayed recovery.

This study employs a two-stage design: First, based on a prospective clinical cohort, we use Growth Mixture Modeling to identify recovery trajectory subgroups, and then employ LPA, network analysis, and exploratory SEM to characterize baseline immuno-metabolic profiles and examine hypothesized associative pathways; second, we use survey-weighted NHANES data to assess whether the DII-low back pain association is corroborated at the population level. This study aims to provide lifestyle-based biological clues for the early identification of patients at risk for LBP chronification.

## 2 Materials and methods

### 2.1 Study design and population

This study adopted a two-stage design, comprising a prospective single-center clinical cohort study and a large-scale population-based cross-sectional study.

This prospective longitudinal cohort study was conducted at the Department of Conservative Treatment, Luoyang Orthopedic-Traumatological Hospital of Henan Province (Henan Provincial Orthopedic Hospital), between January 2024 and March 2025. The study protocol was approved by the Ethics Committee (2024YJSKT0008-01) and strictly adhered to the Declaration of Helsinki. Patients were enrolled based on the following inclusion criteria: (1) a confirmed clinical diagnosis of LDH by an orthopedic surgeon using MRI or CT imaging; (2) presentation of typical LDH symptoms, such as low back pain, radiating leg pain, or signs of nerve root compression; (3) age ≥ 18 years; (4) symptom duration ≥ 3 months to exclude acute phase cases; and (5) the ability to comprehend and complete required questionnaires (e.g., dietary assessments, psychological scales) along with the provision of voluntary informed consent.

Exclusion criteria were: (1) Severe systemic diseases, such as malignancy, severe cardiovascular disease (e.g., heart failure), or severe hepatic/renal insufficiency (affecting metabolic and inflammatory markers); (2) Psychiatric or neurological disorders, such as schizophrenia, severe depression (requiring medication), dementia, or epilepsy, to avoid interference with emotional assessment; (3) Spinal surgery or major trauma within the past 6 months, which may affect inflammatory markers; (4) Specific medication use: long-term use of immunosuppressants, corticosteroids, antidepressants, or NSAIDs (potential interference with inflammation and mood assessment); (5) Specific dietary patterns: e.g., strict ketogenic diet, very low-carbohydrate diet, or long-term fasting (significantly affecting gut microbiota and inflammatory status); (6) Pregnant or lactating women: hormonal changes may affect inflammation and mood status; (7) Night shift workers, as standard Eating Jetlag cannot be calculated for this group; (8) Incomplete data or loss to follow-up: inability to complete dietary records, inflammatory testing, or psychological assessment. We acknowledge that the exclusion of patients using long-term NSAIDs may limit generalizability, as NSAIDs are a common component of conservative LDH management. This criterion was applied to minimize confounding of baseline inflammatory markers (IL-6), which served as a core variable. Similarly, the exclusion of patients with severe depression requiring medication was intended to reduce confounding of sleep and pain assessments, though this may introduce a ceiling effect on mood-related outcomes. These impacts on external validity are further addressed in the Limitations section. A total of 391 patients were initially enrolled, and 324 completed the 6-month follow-up (completion rate 82.9%). Baseline comparisons between completers and non-completers are provided in Supplementary Table 1. As this was a consecutive observational clinical cohort, no formal *a priori* power calculation was performed; however, the final sample of 324 with a smallest trajectory class of *n* = 65 (20.1%) is consistent with simulation-based recommendations for GMM with three time points.

### 2.2 Dietary and Chrono-nutritional assessment

Dietary Inflammatory Index (DII): Dietary data were collected using a validated semi-quantitative Food Frequency Questionnaire (FFQ) (10). Based on the calculation framework established by Shivappa et al. (11), energy-adjusted DII scores were calculated using 28 of the 45 possible nutrients and food components available from our FFQ. The complete list of included and unavailable parameters is provided in Supplementary Table 2. Positive values indicate a more pro-inflammatory diet, whereas negative values indicate a more anti-inflammatory diet. Eating Jetlag (EJL): Detailed meal schedules for workdays and free days were collected. Dietary data acquisition followed standardized procedures using a continuous non-consecutive 3-day 24-h dietary recall method, including two workdays and one freeday. Workdays were defined as participants’ usual working or routine weekdays within a typical week, whereas freedays referred to weekly rest days; this information was self-reported during the dietary interview. Participants were required to report the types, quantities, and timing of all foods and beverages consumed in the previous 24 h. Only caloric foods and beverages were counted as eating events; water and non-caloric beverages were not considered. Any reported caloric intake, regardless of portion size, was treated as an eating event. If eating occurred after midnight but before the main sleep episode, it was classified as the last eating event of the previous day. EJL was defined as the absolute difference in eating midpoint between workdays and freedays.

Data collection was performed by trained researchers via face-to-face interviews or telephone follow-ups. Participants were required to report in detail the types, quantities, and specific ingestion time points of all food and beverages consumed in the past 24 h. To minimize recall bias, for patients with cognitive or memory difficulties, immediate family members or primary caregivers assisted in confirming dietary information.

Eating window definition: Only caloric foods and beverages were counted as eating events; water and non-caloric beverages were excluded. The eating window started at the time of the first bite of food or calorie-containing beverage after waking and ended at the time of the last caloric intake before nocturnal sleep. Any caloric intake, regardless of portion size (including late-night snacks), was treated as an eating event. Cross-day eating determination: If eating occurred after midnight due to delayed bedtime (i.e., before the main sleep phase), it was classified as the last eating event of the previous day. EJL is defined as the absolute difference in the eating midpoint (the midpoint between the first and last eating event) between workdays and free days. The calculation formulas are Equations 1, 2:


$$\text{Eating Midpoint}=\frac{\text{First Meal Timing}+\text{Last Meal Timing}}{2}$$ (1)

$$\mathrm{EJL}=|\text{Eating Midpoin}t_{\text{Free days}}-\text{Eating Midpoin}t_{\text{Work days}}|$$ (2)

### 2.3 Clinical outcomes and biochemical indicators

Longitudinal Follow-up: Oswestry Disability Index (ODI) scores and Visual Analog Scale (VAS) scores for pain were collected at baseline, 3 months, and 6 months post-treatment. Inflammatory Markers: Venous blood was collected in the fasting state on the morning of baseline. Serum Interleukin-6 (IL-6) levels were determined using Enzyme-Linked Immunosorbent Assay (ELISA) as the core biomarker of systemic inflammation. Smoking and drinking status were self-reported and coded as yes/no. Hypertension and diabetes were recorded according to self-reported physician diagnosis or current treatment and coded as yes/no. HbA1c and CRP were collected as baseline biochemical covariates from fasting blood samples.

### 2.4 Statistical analysis strategy

All statistical analyses were performed in the R software environment (version 4.3.1). The two-sided significance level was set at *α* = 0.05. To identify heterogeneity patterns of functional recovery during conservative treatment in a data-driven manner, we analyzed ODI scores at baseline, 3 months, and 6 months. All patients received standardized conservative treatment at our institution, including physical therapy, pharmacotherapy (excluding long-term NSAIDs per exclusion criteria), and rehabilitation guidance.

#### 2.4.1 Identification of longitudinal recovery trajectories

Growth Mixture Modeling (GMM) was performed using the lcmm R package. Models with 1 to 5 latent classes were fitted and compared using log-likelihood, AIC, BIC, SABIC, entropy, and smallest class proportion. Given that only three ODI measurement occasions were available (baseline, 3 months, 6 months), only linear trajectories could be estimated, and the model was kept parsimonious. Detailed fit statistics for all candidate models are provided in Supplementary Table 3. The final model was selected based on overall fit, convergence, minimum class size (>5%), and clinical interpretability. Patients were classified into subgroups labeled “Rapid Recovery,” “Delayed Recovery,” and “Chronic Persistent” according to their observed ODI patterns.

#### 2.4.2 Risk feature profiling and baseline comparison

Latent Profile Analysis (LPA): To explore baseline immuno-metabolic heterogeneity, we performed exploratory LPA using standardized baseline DII, EJL, IL-6, BMI, sleep score, and pain VAS (using the tidyLPA R package). We acknowledge that using the same variables for clustering and subsequent between-group comparison is inherently descriptive; therefore, LPA results were interpreted as data-driven profiling rather than independent discovery. Detailed fit indices (AIC, BIC, entropy, BLRT), class proportions, and cross-tabulation with GMM trajectory groups are provided in Supplementary Table 4. Baseline Difference Analysis: After determining GMM trajectory groups, One-way ANOVA, Kruskal-Wallis H test, or Chi-square test were used to compare demographic characteristics and clinical baseline parameters among different subgroups. Collinearity and Correlation Visualization: Spearman rank correlation coefficients were calculated for core continuous variables. A correlation heatmap was plotted, displaying the correlation matrix with variable order determined by hierarchical clustering.

#### 2.4.3 Exploration of non-linear dose–response relationships

Restricted Cubic Splines (RCS) models (using the rms R package) were employed to examine the form of association between DII and poor recovery (defined as 6-month ODI > 20%), after adjusting for age, sex, BMI, EJL, and baseline ODI. The model was set with 3 knots. Wald tests were used to assess the overall association and the nonlinear component separately. Detailed test statistics are provided in Supplementary Table 5. Because the nonlinear component was not statistically significant, no threshold-based categorization was applied in the primary interpretation.

#### 2.4.4 Symptom-mechanism network analysis

To explore the partial-correlation structure among lifestyle, inflammatory, and clinical symptom variables, a Gaussian Graphical Model (GGM) was constructed (using the qgraph R package).

Network Construction: Nodes included DII, EJL, IL-6, BMI, Age, Sleep Disturbance, Pain Intensity, and ODI; Edges represented partial correlation coefficients after controlling for all other nodes. Regularization: The Graphical LASSO algorithm combined with the Extended Bayesian Information Criterion (EBICglasso) was used for regularization to shrink weak or spurious partial correlations to zero, thereby obtaining a sparse and robust network structure. Core Indicators: Strength centrality of nodes was calculated to identify core symptoms or mechanism nodes with the greatest influence in the network. Additionally, non-parametric Bootstrap methods (1,000 resamples) were used to assess the stability of the network structure and the accuracy of centrality indices (using the bootnet R package).

#### 2.4.5 Path analysis and causal mediation

Based on the topological structure revealed by network analysis, exploratory Structural Equation Modeling (SEM) was performed (using the lavaan R package) to examine hypothesized baseline associations among DII, EJL, IL-6, sleep score, pain VAS, and baseline ODI. Estimation Method: Maximum Likelihood Estimation. Model fit was evaluated using the Comparative Fit Index (CFI), Root Mean Square Error of Approximation (RMSEA), and Standardized Root Mean Square Residual (SRMR). Detailed fit indices and standardized path coefficients are provided in Supplementary Table 6. Given the observational design, the single-time measurement of IL-6, and the fact that all predictor variables were measured concurrently at baseline, the SEM results were interpreted as hypothesis-generating associative pathways rather than confirmatory causal mediation.

#### 2.4.6 Prediction model construction and external validation

Nomogram Construction: Based on a multivariable logistic regression model, a nomogram was constructed as an internal visualization tool for individualized risk assessment. The underlying univariable and multivariable logistic regression results are provided in Supplementary Table 7.

Model Evaluation: Calibration curves were used to assess the consistency between predicted probabilities and actual incidence (verified by Bootstrap resampling); Decision Curve Analysis (DCA) was employed to evaluate the clinical net benefit. Because this model was evaluated only internally (calibration and DCA), it should not be considered externally validated.

NHANES population-level corroboration: Weighted data from the 2009–2010, 2015–2016, and 2017–2018 NHANES cycles were used as a population-level corroboration dataset; the detailed selection flow is shown in Supplementary Figure 1. The Day 1 dietary sample weight (WTDRD1) was divided by 3 to create combined survey weights across three cycles, and SDMVSTRA and SDMVPSU were used as the stratification and primary sampling unit variables, respectively. Survey-weighted logistic regression and survey-weighted RCS models were fitted using the survey R package. The survey design specification is summarized in Supplementary Table 8. Because NHANES is cross-sectional and evaluates prevalent low back pain rather than longitudinal recovery trajectories, these analyses were interpreted as population-level corroboration rather than formal external validation of the prognostic model.

## 3 Results

### 3.1 Longitudinal heterogeneity trajectories of functional recovery under conservative treatment

**Figure 1:**
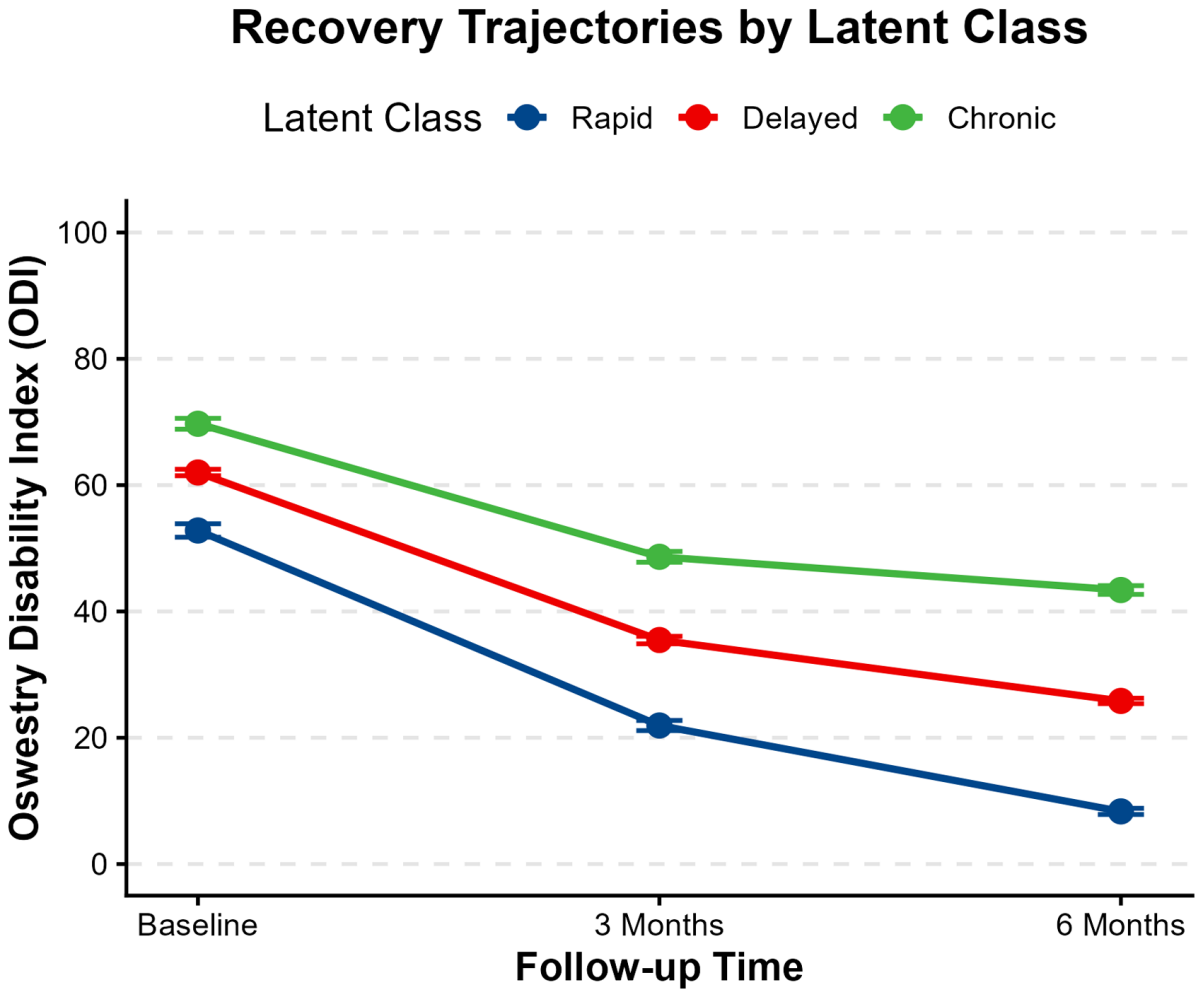
GMM-derived ODI trajectories over 6 months: Rapid Recovery (*N* = 65, green), Delayed Recovery (*N* = 182, orange), and Chronic Persistent (*N* = 77, purple). The *y*-axis shows ODI scores.

To characterize heterogeneity in functional recovery under conservative treatment, we performed Growth Mixture Modeling (GMM) using longitudinal ODI data at baseline, 3 months, and 6 months. By comparing 1-to-5-class solutions (Supplementary Table S3), we selected a three-class model based on overall fit, convergence, class size, and clinical interpretability (Figure 1).

The first trajectory was defined as the “Rapid Recovery Group” (*N* = 65, 20.1%). These patients had the lowest baseline ODI among the three groups and showed a steep decline in ODI scores within the first 3 months, approaching near-remission levels by 6 months. The second trajectory was the “Delayed Recovery Group” (*N* = 182, 56.2%), characterized by intermediate baseline disability and gradual improvement over follow-up. The third trajectory was termed the “Chronic Persistent Group” (*N* = 77, 23.8%). Patients in this group had the highest baseline functional disability and showed limited decline in ODI scores throughout the follow-up period. Timepoint-specific ODI descriptive statistics are provided in Supplementary Table 9.

As shown in Supplementary Figures 2A,B, model diagnostic charts supported the classification. The normal Q-Q plot of subject-specific residuals showed data points distributed closely around the diagonal, indicating that model assumptions were reasonably met. Given that only three time points were available, the trajectory shapes should be interpreted as linear trends rather than complex nonlinear patterns. This prognostic differentiation suggests that, in addition to structural pathological changes, intrinsic patient characteristics may contribute to the process of disease chronification.

### 3.2 Identification of Immuno-metabolic risk phenotypes and baseline profiling

**Figure 2:**
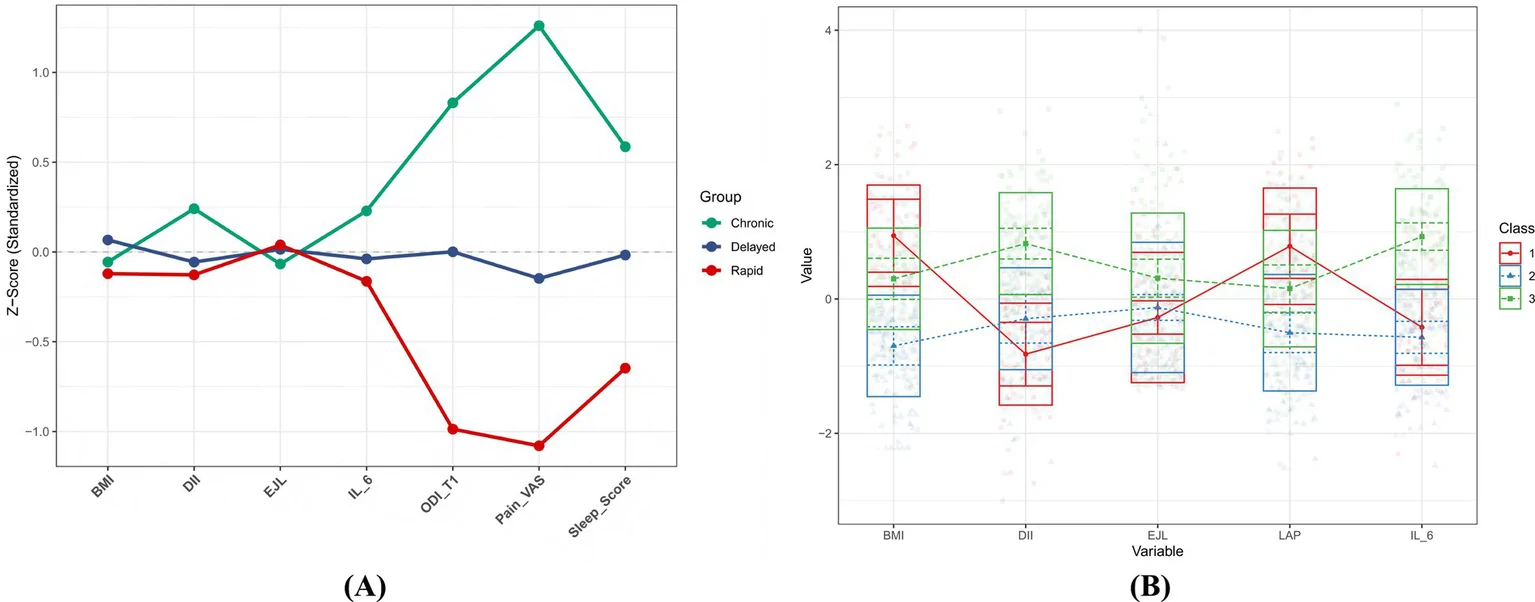
Exploratory Latent Profile Analysis (LPA) of baseline immuno-metabolic features. **(A)** Parallel coordinate plot of standardized variable means for three LPA-derived profiles. **(B)** Cross-tabulation between LPA profiles and GMM trajectory groups. Profiles are data-driven groupings; they do not correspond one-to-one with GMM trajectory classes.

To investigate baseline heterogeneity related to diet, rhythm, inflammation, and symptoms, we performed exploratory LPA (Figures 2A,B; Supplementary Table 4). The resulting profiles differed descriptively in DII, EJL, IL-6, and symptom burden. Because the same variables were used for profiling and subsequent comparison, and because fit indices were not fully concordant across candidate solutions, these profiles were interpreted as data-driven descriptions rather than independently validated phenotypes.

Baseline differences across the three GMM trajectory groups are summarized in Table 1. There were no significant differences among the three groups in age, sex, BMI, smoking, drinking, hypertension, diabetes, HbA1c, EJL, dietary rhythm regularity, lipid accumulation product (LAP), or CRP (all *p* > 0.05). However, compared with the Rapid Recovery group, the Chronic Persistent group had significantly higher DII (0.85 ± 1.45 vs. 0.31 ± 1.41, *p* = 0.030), higher IL-6 (5.99 ± 1.06 vs. 5.56 ± 1.17, *p* = 0.021), worse sleep score (11.55 ± 3.35 vs. 7.22 ± 3.92, *p* < 0.001), higher pain VAS (6.34 ± 1.96 vs. 1.00 ± 0.95, *p* < 0.001), and higher baseline ODI (69.71 ± 7.57 vs. 52.82 ± 8.57, *p* < 0.001). These findings suggest that prognostic heterogeneity was associated with baseline inflammatory and symptom-level characteristics rather than demographic or metabolic differences.

**Table 1:** Baseline characteristics.

Characteristic	Overall (*N* = 324)	Chronic persistent (*N* = 77)	Delayed recovery (*N* = 182)	Rapid recovery (*N* = 65)	*p* value
Demographics					
Age, years	54.9 (9.9)	55.1 (9.9)	55.0 (9.3)	54.2 (11.5)	0.905
Sex, female	192 (59%)	46 (60%)	105 (58%)	41 (63%)	0.700
BMI, kg/m^2^	26.1 (3.4)	25.9 (3.6)	26.3 (3.4)	25.7 (3.2)	0.400
Lifestyle					
Smoking, yes	72 (22%)	16 (21%)	43 (24%)	13 (20%)	0.800
Drinking, yes	90 (28%)	20 (26%)	54 (30%)	16 (25%)	0.700
Dietary rhythm regular, yes	239 (74%)	51 (66%)	134 (74%)	54 (83%)	0.075
Comorbidities					
Hypertension, yes	205 (63%)	48 (62%)	122 (67%)	35 (54%)	0.200
Diabetes, yes	162 (50%)	34 (44%)	95 (52%)	33 (51%)	0.500
Dietary and chrono-nutritional					
DII score	0.50 (1.48)	0.85 (1.45)	0.41 (1.47)	0.31 (1.41)	0.030
EJL, hours	0.80 (0.59)	0.76 (0.54)	0.81 (0.62)	0.83 (0.57)	0.800
Biochemical markers					
IL-6, pg./mL	5.74 (1.10)	5.99 (1.06)	5.70 (1.08)	5.56 (1.17)	0.021
CRP, mg/L	3.32 (0.71)	3.40 (0.71)	3.32 (0.69)	3.18 (0.78)	0.200
HbA1c, %	7.14 (1.83)	6.79 (1.65)	7.25 (1.88)	7.24 (1.87)	0.200
LAP	60.30 (13.05)	58.99 (12.82)	60.34 (13.57)	62.24 (11.15)	0.200
Clinical symptoms					
Sleep score (PSQI)	9.50 (3.50)	11.55 (3.35)	9.43 (2.86)	7.22 (3.92)	<0.001
Pain VAS	3.50 (2.30)	6.34 (1.96)	3.13 (1.24)	1.00 (0.95)	<0.001
Baseline ODI	62.0 (9.3)	69.71 (7.57)	62.00 (6.97)	52.82 (8.57)	<0.001

Values are mean (SD) or *n* (%). *p* values from Kruskal-Wallis rank sum test (continuous) or Pearson’s chi-square test (categorical). DII, Dietary Inflammatory Index; EJL, Eating Jetlag; IL-6, interleukin-6; CRP, C-reactive protein; HbA1c, glycated hemoglobin; LAP, lipid accumulation product; PSQI, Pittsburgh Sleep Quality Index; VAS, Visual Analog Scale; ODI, Oswestry Disability Index. Smoking and drinking status were self-reported (yes/no). Hypertension and diabetes were recorded according to self-reported physician diagnosis or current treatment.

**Figure 3:**
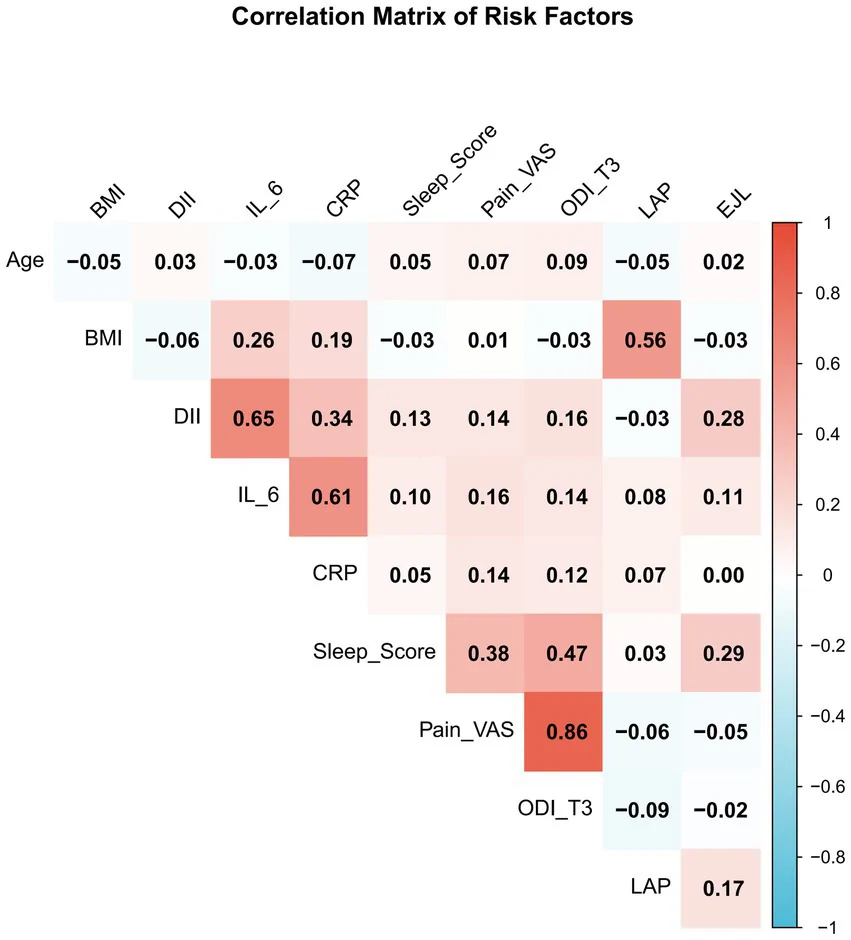
Correlation matrix of risk factors and symptoms.

The correlation heatmap (Figure 3) showed that DII clustered more closely with inflammatory markers, whereas EJL clustered more closely with symptom-related variables such as sleep score and pain VAS. These descriptive associations informed the subsequent exploratory network and SEM analyses.

### 3.3 Non-linear dose–response relationship between Dietary Inflammatory Index and chronification risk

**Figure 4:**
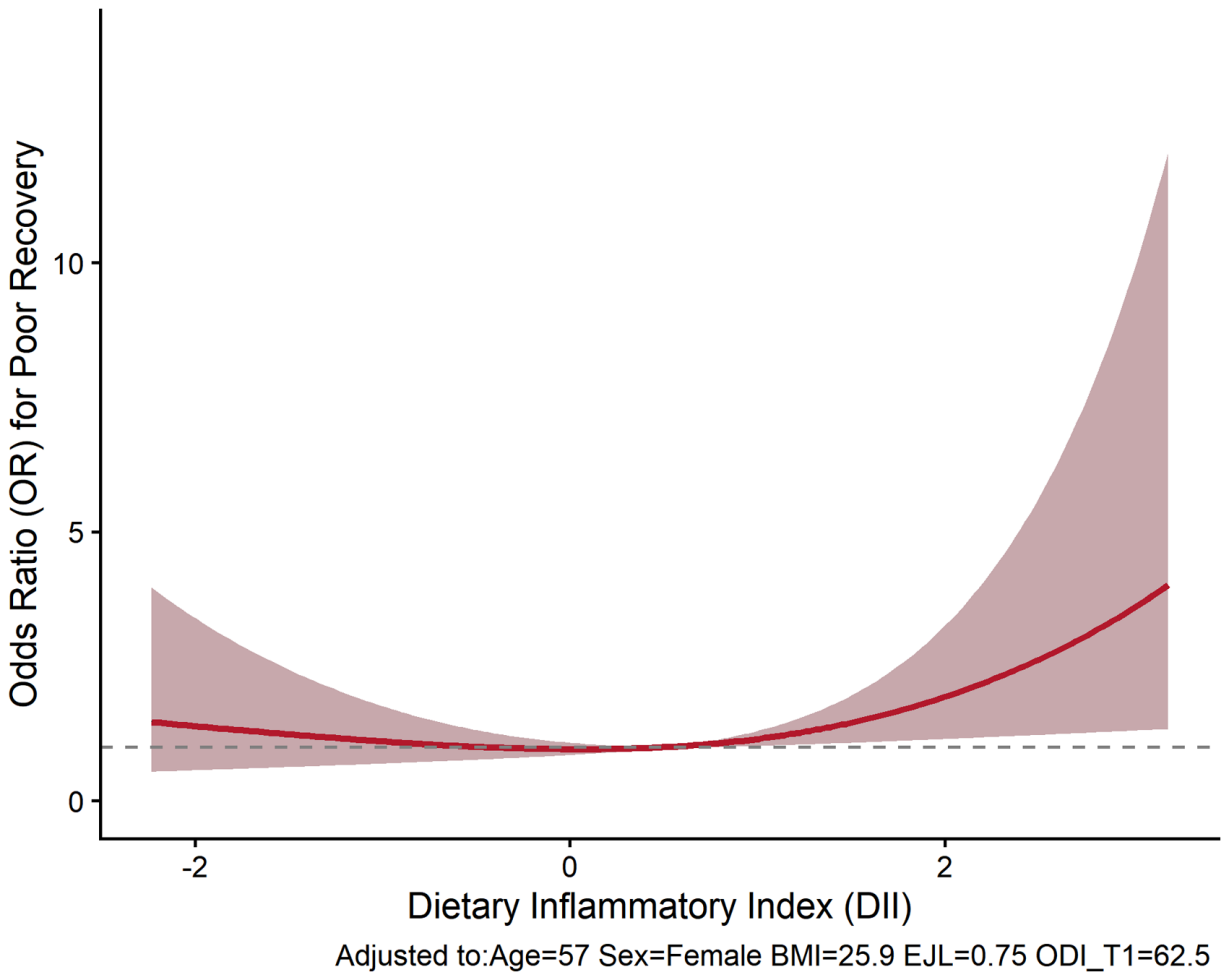
Restricted cubic spline analysis of DII and risk of chronic trajectory.

To examine the shape of the association between dietary inflammatory load and prognosis, we fitted a Restricted Cubic Spline (RCS) model for DII and poor recovery, adjusted for age, sex, BMI, EJL, and baseline ODI (Figure 4; Supplementary Table 5).

The overall association was statistically significant (*p* = 0.018), whereas the nonlinear component was not significant (*p* = 0.333). Thus, the data supported an overall adverse association between higher DII and poor recovery, but did not confirm a J-shaped or threshold relationship. The spline curve showed a generally monotonic upward trend, with the confidence interval widening at extreme DII values due to sparse data. These results indicate that dietary inflammatory load is associated with chronification risk in an approximately dose-dependent manner, though the limited event count (n = 77 chronic cases) warrants cautious interpretation of the curve shape at the extremes.

### 3.4 Multi-dimensional interaction network of symptoms-mechanisms and causal path analysis

**Figure 5:**
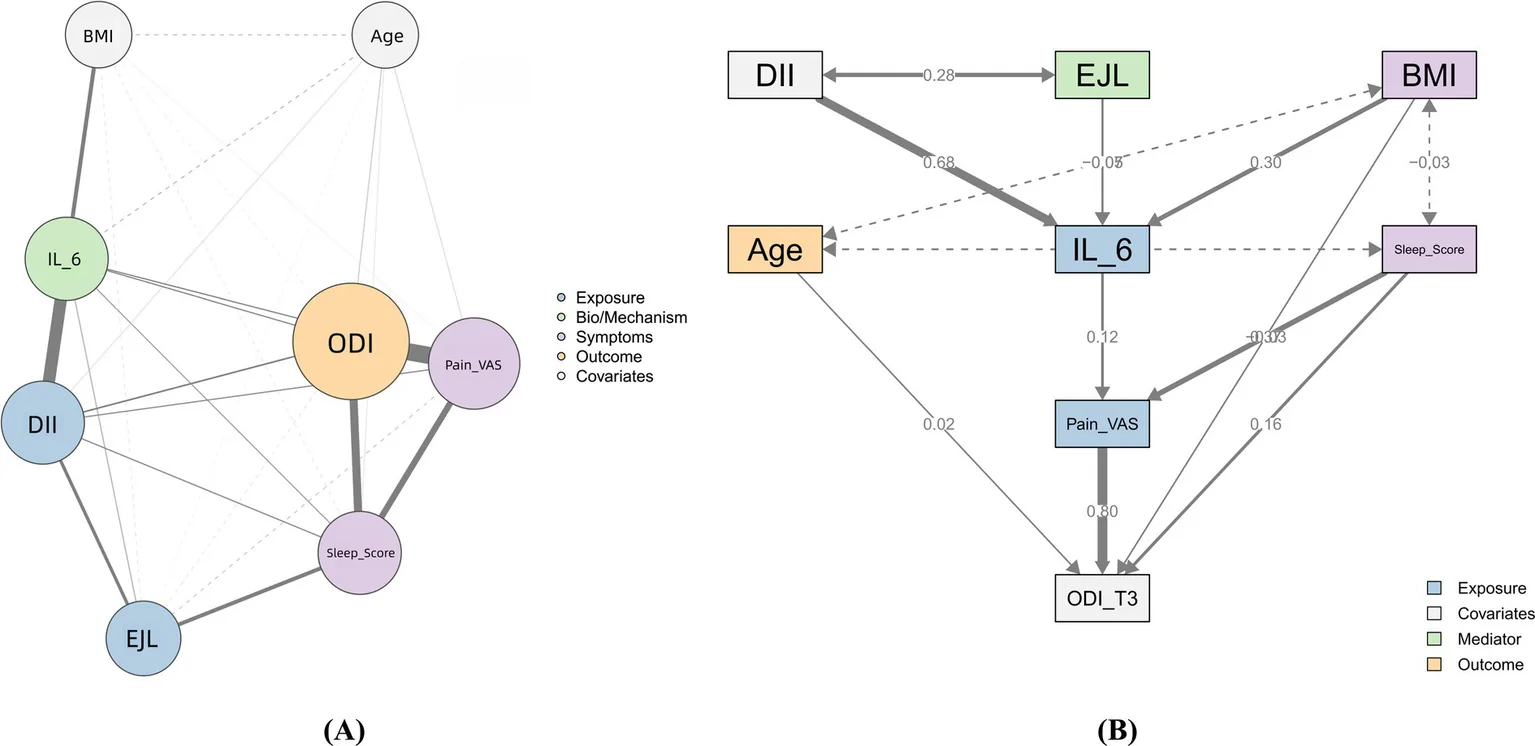
Network architecture and path analysis of symptom-mechanism interactions. **(A)** Topological architecture of the symptom-mechanism network. **(B)** Path analysis of the “diet-inflammation-symptom” cascade.

To explore the partial-correlation structure among lifestyle, inflammatory, and clinical symptom variables, we constructed a Gaussian Graphical Model (Figure 5A) and performed exploratory SEM (Figure 5B; Supplementary Table 6).

Network topological analysis revealed that IL-6 occupied a central position connecting upstream exposure factors (DII, EJL) with downstream clinical symptom clusters. BMI and age were located at the periphery of the network, suggesting weaker direct connections with the core diet-inflammation-symptom cluster.

Based on this topology, we performed exploratory SEM to quantify baseline associations. The overall model did not achieve ideal global fit (CFI = 0.865, RMSEA = 0.194, SRMR = 0.060), and therefore the path coefficients were interpreted cautiously. Within this model, DII was strongly positively associated with IL-6 (standardized coefficient = 0.685, *p* < 0.001), and EJL was positively associated with sleep score (standardized coefficient = 0.279, *p* < 0.001). Pain VAS was the strongest proximal correlate of baseline ODI (standardized coefficient = 0.576, *p* < 0.001). The path from IL-6 to pain VAS was marginal and not statistically significant (standardized coefficient = 0.138, *p* = 0.062). The path from sleep score to baseline ODI was not significant (standardized coefficient = 0.033, *p* = 0.530). The direct path from IL-6 to baseline ODI was small, negative, and statistically significant (standardized coefficient = −0.111, *p* = 0.025), which may reflect residual confounding or model misspecification rather than a true protective effect. Accordingly, these SEM findings were interpreted as hypothesis-generating rather than confirmatory evidence of mediation.

### 3.5 Development of an integrated lifestyle-inflammation prognostic model for individualized assessment

**Figure 6:**
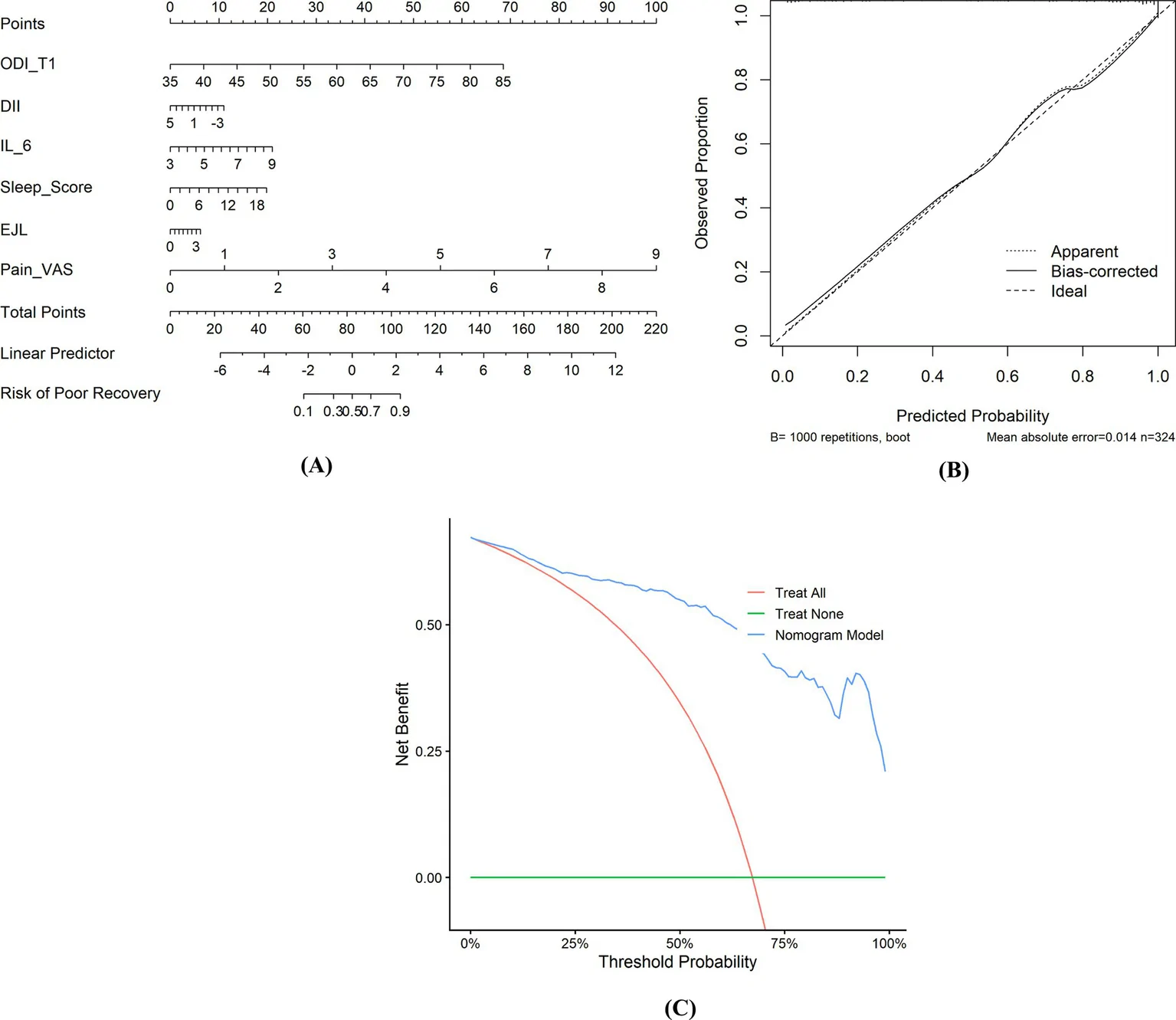
Development and internal evaluation of the immuno-metabolic prognostic nomogram. **(A)** Nomogram integrating baseline ODI, DII, IL-6, sleep score, EJL, and pain VAS. **(B)** Calibration plot (1,000 bootstrap resamples). **(C)** Decision curve analysis.

Based on the multivariable logistic regression model (Supplementary Table 7), we constructed a nomogram integrating baseline ODI, DII, IL-6, sleep score, EJL, and pain VAS as a visualization tool for individualized risk assessment (Figure 6A). In the multivariable model, only sleep score (OR = 1.223, *p* = 0.012), pain VAS (OR = 3.456, *p* < 0.001), and baseline ODI (OR = 1.153, p < 0.001) retained independent significance. DII (OR = 1.256, *p* = 0.317), EJL (OR = 0.648, *p* = 0.307), and IL-6 (OR = 0.929, *p* = 0.793) were not independently significant after mutual adjustment, suggesting that their prognostic relevance may operate through shared variance with symptom-level variables rather than as independent predictors.

The calibration curve (Figure 6B) showed adequate agreement between predicted probabilities and observed incidence (Mean Absolute Error = 0.021). Decision Curve Analysis (DCA, Figure 6C) indicated that the model provided net benefit over default “Treat-all” or “Treat-none” strategies across a threshold probability range of approximately 5 to 85%. Because this model was evaluated only internally via bootstrap resampling, its performance in external populations remains to be established.

### 3.6 Population-level association analysis of dietary inflammatory potential and low Back pain risk

Using survey-weighted NHANES data (2009–2010, 2015–2016, 2017–2018; Supplementary Table 8), we assessed whether the DII-low back pain association observed in the clinical cohort was corroborated at the population level. Baseline characteristics of the NHANES analytic sample are presented in Supplementary Table 10. Participants in the highest DII quartile (Q4) had higher hs-CRP levels and longer Eating Jetlag compared with those in the lowest quartile (Q4 vs. Q1: 1.45 vs. 1.13 h, *p* < 0.001), consistent with the co-occurrence of pro-inflammatory dietary patterns and meal-timing irregularity observed in the clinical cohort.

Survey-weighted multivariable logistic regression (Table 2) showed that, after adjusting for age, sex, race/ethnicity, BMI, education, poverty-income ratio, smoking, physical activity, total energy intake, and EJL, participants in the highest DII quartile had significantly higher odds of prevalent low back pain compared with the lowest quartile (OR = 1.56, 95% CI: 1.28–1.91, *p* < 0.001).

**Table 2:** Survey-weighted logistic regression of DII and prevalent low back pain.

Characteristic	Crude Model OR (95% CI)	*p*	Model 1 OR (95% CI)	*p*	Model 2 OR (95% CI)	*p*	Model 3 OR (95% CI)	*p*
DII (Continuous)	1.08 (1.05–1.10)	<0.001	1.07 (1.04–1.09)	<0.001	1.05 (1.02–1.08)	<0.001	1.08 (1.05–1.12)	<0.001
DII Quartiles								
Q1 (Ref)	Ref	—	Ref	—	Ref	—	Ref	—
Q2	1.30 (1.11–1.52)	0.001	1.29 (1.10–1.52)	0.002	1.24 (1.05–1.46)	0.011	1.31 (1.10–1.55)	0.002
Q3	1.41 (1.21–1.65)	<0.001	1.35 (1.15–1.58)	<0.001	1.27 (1.08–1.50)	0.004	1.40 (1.17–1.68)	<0.001
Q4	1.58 (1.36–1.84)	<0.001	1.47 (1.25–1.73)	<0.001	1.35 (1.15–1.59)	<0.001	1.56 (1.28–1.91)	<0.001

Crude Model: unadjusted. Model 1: adjusted for age, sex, race/ethnicity. Model 2: adjusted for Model 1 + BMI, education, PIR, smoking, physical activity. Model 3: adjusted for Model 2 + total energy intake, EJL.

**Figure 7:**
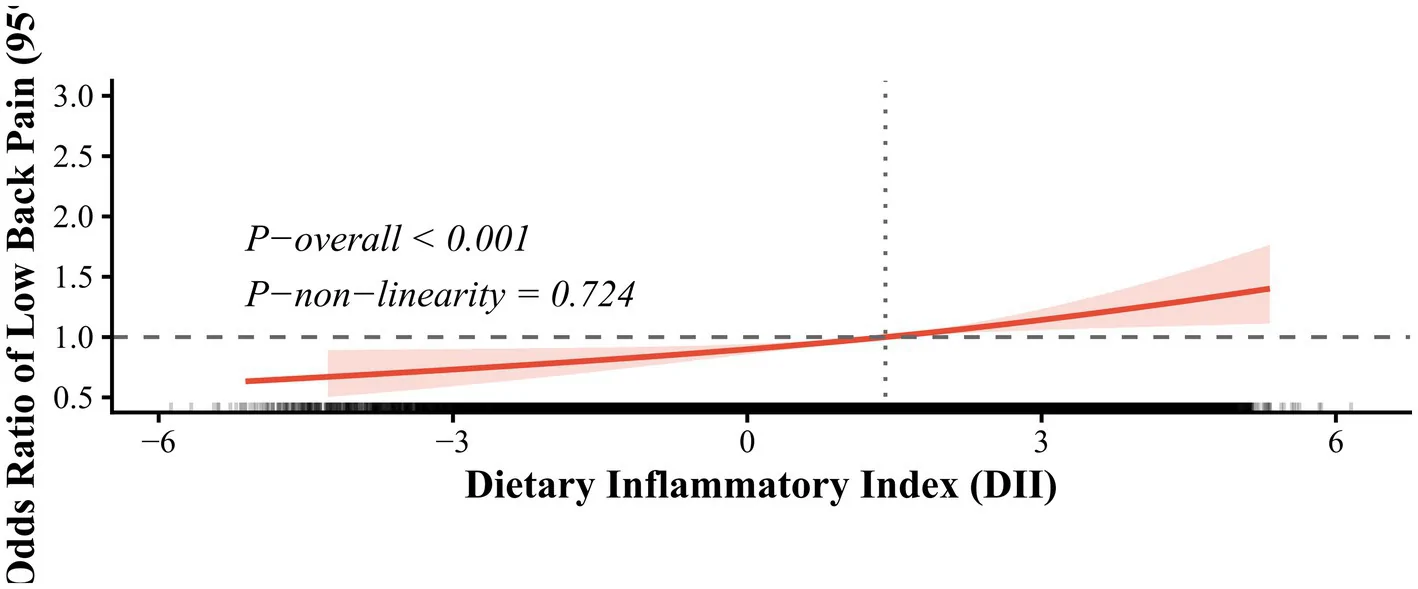
Survey-weighted RCS analysis of DII and prevalent low back pain in NHANES. Solid line: adjusted OR; shaded area: 95% CI. Adjusted for age, sex, race/ethnicity, BMI, education, PIR, smoking, physical activity, total energy intake, and EJL. P-overall < 0.001; P-non-linearity = 0.724.

The survey-weighted RCS model (Figure 7) showed a significant overall association between DII and LBP risk (P-overall < 0.001), with the nonlinear component not significant (P-non-linearity = 0.724), indicating an approximately linear dose–response relationship. Notably, this linear pattern in the general population differs from the spline shape observed in the clinical cohort, where the overall association was significant but the curve appeared steeper at higher DII values. This discrepancy may reflect differences in population characteristics, outcome definitions (prevalent LBP vs. longitudinal recovery trajectory), and sample size.

## 4 Discussion

### 4.1 Longitudinal association of diet, rhythm, and LDH recovery outcomes

Through a prospective cohort and multi-dimensional statistical modeling, this study provides preliminary evidence that chrono-nutritional misalignment and pro-inflammatory dietary load are associated with less favorable recovery outcomes following conservative treatment for LDH. Our core finding identifies significant heterogeneity in functional recovery: approximately one-quarter of patients were classified in a Chronic Persistent trajectory. This subgroup exhibited higher baseline DII, higher IL-6, and more severe sleep and pain scores compared with the Rapid Recovery group, whereas demographic and metabolic variables did not differ significantly. These findings suggest that the organism’s systemic inflammatory and behavioral milieu may contribute to recovery heterogeneity beyond what anatomical structures alone would predict, and provide observational evidence supporting the relevance of lifestyle-immunity-pain interactions in LDH prognosis.

### 4.2 Pro-inflammatory diet and Eating Jetlag

Diet, as a modifiable factor regulating systemic inflammation, is often underappreciated in pain management. Our RCS analysis showed a significant overall association between DII and poor recovery (*p* = 0.018), though the nonlinear component was not statistically significant (*p* = 0.333). Thus, the data support an approximately dose-dependent adverse association rather than a clear threshold effect. This finding is consistent with the NHANES analysis, where the DII-LBP association was also approximately linear (P-non-linearity = 0.724), suggesting that the relationship between dietary inflammatory load and pain-related outcomes may be continuous rather than threshold-dependent across different populations and outcome definitions.

Mechanistically, a high-DII diet (rich in saturated fats and refined sugars, lacking anti-inflammatory micronutrients) can induce increased intestinal barrier permeability, leading to lipopolysaccharide (LPS) translocation into the blood, triggering “metabolic endotoxemia” (12). This sustained low-grade inflammatory state can activate glial cells in the dorsal root ganglia (DRG), releasing pro-inflammatory cytokines (e.g., IL-6), thereby lowering the threshold of nociceptors (13). In our cohort, the Chronic Persistent group had higher baseline DII and IL-6 compared with the Rapid Recovery group, which is consistent with the hypothesis that a pro-inflammatory diet may contribute to maintaining a peripheral sensitized state, though the observational design precludes causal inference.

Another contribution of this study is the incorporation of a chrono-nutrition perspective, examining the association of Eating Jetlag (EJL) with LDH prognosis (14, 15). In our exploratory LPA, higher EJL tended to co-occur with higher DII, consistent with the notion that dietary composition and timing irregularity may cluster as part of a broader lifestyle pattern. Chronobiology suggests that meal timing is a primary Zeitgeber (time-giver) for peripheral clocks in tissues like the liver and gut (16). When eating patterns fluctuate substantially between workdays and free days (i.e., increased EJL), peripheral clocks may desynchronize from the central master clock in the suprachiasmatic nucleus (SCN) (17). This molecular disruption of circadian rhythms has been shown to attenuate the anti-inflammatory rhythm of glucocorticoids and upregulate the NF-κB pathway (18–20). In our network analysis, EJL showed partial correlations with both IL-6 and sleep quality, consistent with this hypothesis. However, EJL was not independently associated with poor recovery in the multivariable logistic regression (OR = 0.648, *p* = 0.307), suggesting that its prognostic relevance may be indirect or confounded by other variables.

### 4.3 Joint pathways of the symptom-mechanism network

Previous studies often regarded inflammation as a concomitant product of tissue injury. Our exploratory SEM examined whether IL-6 was associated with functional disability directly or through symptom-level variables. The strongest path in the model was DII → IL-6 (standardized coefficient = 0.685), and the strongest proximal correlate of baseline ODI was pain VAS (standardized coefficient = 0.576). The path from IL-6 to pain VAS was marginal (*p* = 0.062), and the path from sleep score to ODI was not significant (*p* = 0.530). These findings are broadly consistent with the hypothesis that systemic inflammation may relate to disability through symptom amplification (21, 22), but the suboptimal global model fit (CFI = 0.865, RMSEA = 0.194) and the cross-sectional nature of the baseline measurements preclude definitive conclusions about mediation or temporal ordering.

Importantly, all predictor variables in the SEM were measured concurrently at baseline. IL-6 was measured only once, and we did not capture dynamic inflammatory changes during follow-up. Therefore, the observed associations should be understood as baseline cross-sectional relationships rather than longitudinal causal pathways. Future studies with repeated inflammatory measurements would be needed to test whether changes in IL-6 over time mediate the relationship between dietary patterns and functional recovery.

### 4.4 Macro-validation and clinical translation

To assess whether the DII-low back pain association observed in the clinical cohort was corroborated at the population level, we analyzed survey-weighted NHANES data. The NHANES analysis confirmed a significant, approximately linear association between DII and prevalent low back pain (OR = 1.56, *p* < 0.001) (23). However, it is important to note that the NHANES analysis and the clinical cohort differ fundamentally in design (cross-sectional vs. longitudinal), population (general U. S. population vs. Chinese LDH patients), and outcome (prevalent LBP vs. recovery trajectory). Therefore, the NHANES results should be interpreted as population-level corroboration of the DII-LBP association rather than formal external validation of the prognostic model or the longitudinal findings.

The nomogram we constructed integrates lifestyle, inflammatory, and clinical variables as a visualization tool for individualized risk assessment. However, in the multivariable model, only sleep score, pain VAS, and baseline ODI retained independent significance, while DII, EJL, and IL-6 did not. This suggests that the prognostic contribution of dietary and inflammatory variables may be largely captured by symptom-level measures. Nonetheless, from a clinical perspective, dietary patterns and meal timing regularity represent modifiable upstream factors that may influence downstream (24, 25). For patients identified as having high dietary inflammatory load and irregular meal timing, targeted anti-inflammatory dietary guidance (e.g., increasing dietary fiber and polyphenol intake) and chrono-nutritional counseling (e.g., regularizing eating windows) may represent low-cost adjunctive strategies worthy of investigation in future interventional trials.

### 4.5 Strengths and limitations

The primary strength of this study lies in the integration of dual perspectives of “chrono-nutrition” and “immunometabolism,” combined with longitudinal clinical cohort and large-scale population data. Utilizing data-driven methods like LPA and network analysis, we explored multi-dimensional lifestyle clustering that traditional linear regression may not capture.

However, several limitations must be considered when interpreting the results. First, causal inference is limited. Although a prospective design was employed, the SEM was based on cross-sectional baseline measurements, and IL-6 was measured only once. The influence of unmeasured confounders (e.g., genetic susceptibility, detailed physical activity intensity, psychological stressors, or specific conservative treatment modalities) cannot be completely ruled out. The observed associations should be understood as statistical relationships rather than definitive biological causality. Second, measurement error exists. Despite using the 3-day 24-h recall method, self-reported dietary data inevitably suffer from recall bias, systematic under-reporting of energy intake, and misreporting of portion sizes. Additionally, the FFQ used in this study has been applied in Chinese clinical populations but has not undergone formal criterion validation against biomarkers in this specific patient group; the reference cited in the original submission (10) was incorrect and has been replaced. The DII was calculated from 28 of 45 possible parameters, which may result in a narrower score range and attenuated associations compared with studies using more complete parameter sets. Third, the indirectness of the mechanism. We speculate that EJL affects inflammation by disrupting peripheral clock genes (e.g., Clock or Bmal1), but this study did not collect tissue samples to directly measure the expression of these genes; this mechanistic hypothesis requires further verification through basic experiments. Fourth, external validity. The clinical cohort was a single-center study with a relatively limited sample size (N = 324) and primarily an Asian population. The smallest trajectory class (Rapid Recovery, N = 65) limits the precision of between-group comparisons. Although NHANES provided population-level corroboration, its cross-sectional nature and different outcome definition limit its contribution to prognostic judgment. Fifth, the high prevalence of hypertension (63%) and diabetes (50%) in this cohort is notable and may limit generalizability to younger or healthier LDH populations. This high comorbidity burden may also confound inflammatory markers, as both conditions are independently associated with elevated IL-6 and CRP. Sixth, the GMM was limited to three time points, allowing only linear trajectory estimation. With more frequent assessments, nonlinear recovery patterns might be identifiable. Future multi-center, multi-ethnic longitudinal cohorts with repeated inflammatory measurements and more frequent outcome assessments are needed to validate and extend these findings.

## 5 Conclusion

In summary, this study provides observational evidence that chrono-nutritional misalignment and pro-inflammatory dietary load are associated with less favorable recovery trajectories following conservative treatment for LDH. Exploratory analyses suggest that the co-occurrence of dietary inflammation and meal-timing irregularity may relate to baseline systemic inflammation and symptom burden, though the cross-sectional nature of the baseline measurements and suboptimal SEM fit preclude definitive conclusions about mediation. The DII-low back pain association was corroborated at the population level using NHANES data. These findings support further investigation of dietary and chrono-nutritional factors as modifiable targets in LDH management, and highlight the potential value of incorporating immuno-metabolic and behavioral assessments into prognostic evaluation for degenerative spinal diseases.

## Data Availability

The raw data supporting the conclusions of this article will be made available by the authors, without undue reservation.
