# Supplementary material for: Synergistic impact of chrono-nutritional and pro-inflammatory dietary patterns on chronic pain recovery: a prospective cohort study with population-based corroboration
Source: Front Nutr. 2026 Jul 15;13:1798285. doi: 10.3389/fnut.2026.1798285 (PMC13414752; doi:10.3389/fnut.2026.1798285)
Supplement: Supplementary file 1 [file Table_1.DOCX]

Supplementary Material

**Supplementary Figure 1.** Flowchart of Participant Selection from the NHANES Database.


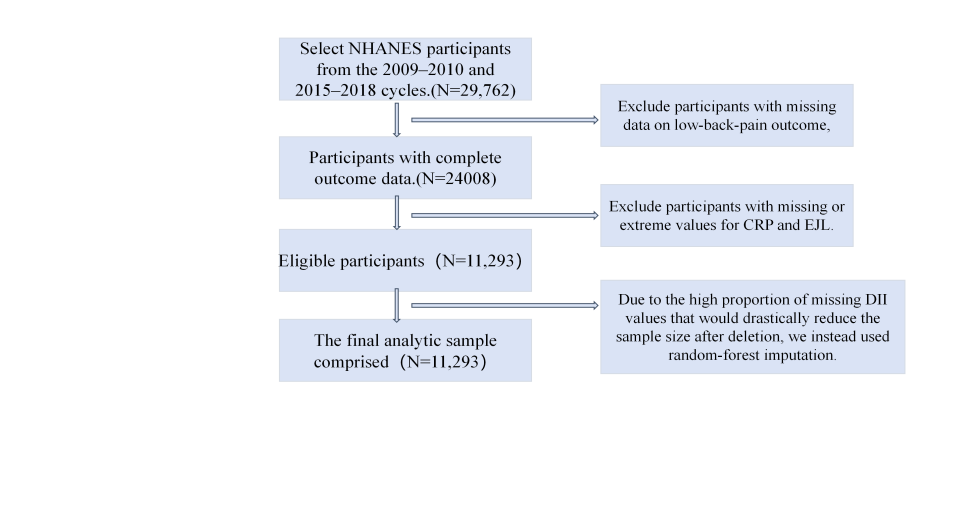


**Supplementary Figure 2.** Diagnostic Evaluation of the Growth Mixture Model.(A) Posterior probability distributions for latent classes; the density clustering near 1.0 indicates high classification certainty.(B) Q-Q plots of marginal and subject-specific residuals, confirming the normality assumption required for the analysis.


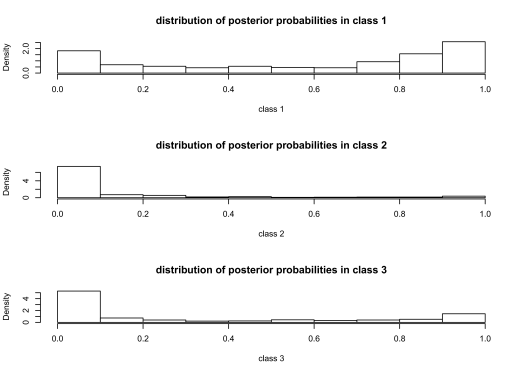
(A)
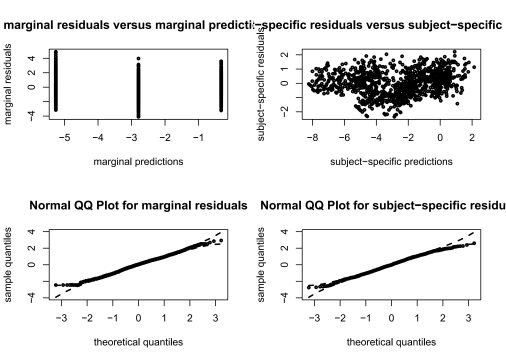
(B)

Supplementary Table 1. Baseline comparison between completers (N = 324) and non-completers (N = 67)

| **Characteristic** | **Completers (N=324)** | **Non-completers (N=67)** | **p-value** |
| --- | --- | --- | --- |
| Age, mean (SD) | 54.9 (9.9) | 53.8 (10.2) | 0.41 |
| Female, n (%) | 192 (59%) | 38 (57%) | 0.72 |
| BMI, mean (SD) | 26.1 (3.4) | 26.4 (3.6) | 0.52 |
| Baseline ODI, mean (SD) | 62.0 (9.3) | 61.3 (9.8) | 0.58 |
| DII, mean (SD) | 0.50 (1.48) | 0.55 (1.52) | 0.80 |
| EJL (h), mean (SD) | 0.80 (0.59) | 0.85 (0.63) | 0.53 |
| IL-6 (pg/mL), mean (SD) | 5.74 (1.10) | 5.80 (1.15) | 0.68 |
| Hypertension, n (%) | 205 (63%) | 44 (66%) | 0.72 |
| Diabetes, n (%) | 162 (50%) | 35 (52%) | 0.76 |

Note: P values from independent-samples t-test (continuous) or chi-square test (categorical). No significant differences were observed, suggesting that attrition was largely non-differential.

Supplementary Table 2. DII food parameters: included and unavailable components

| **NO.** | **Parameter** | **Status** | **Category** |
| --- | --- | --- | --- |
| 1 | Energy (kcal) | Included | Macronutrient |
| 2 | Protein (g) | Included | Macronutrient |
| 3 | Total fat (g) | Included | Macronutrient |
| 4 | Saturated fat (g) | Included | Macronutrient |
| 5 | Carbohydrate (g) | Included | Macronutrient |
| 6 | Fiber (g) | Included | Macronutrient |
| 7 | Cholesterol (mg) | Included | Macronutrient |
| 8 | MUFA (g) | Included | Fatty acid |
| 9 | PUFA (g) | Included | Fatty acid |
| 10 | Omega-3 (g) | Included | Fatty acid |
| 11 | Omega-6 (g) | Included | Fatty acid |
| 12 | Niacin (mg) | Included | Vitamin |
| 13 | Thiamin (mg) | Included | Vitamin |
| 14 | Riboflavin (mg) | Included | Vitamin |
| 15 | Vitamin B6 (mg) | Included | Vitamin |
| 16 | Vitamin B12 (mcg) | Included | Vitamin |
| 17 | Vitamin A (RE) | Included | Vitamin |
| 18 | Vitamin C (mg) | Included | Vitamin |
| 19 | Vitamin D (mcg) | Included | Vitamin |
| 20 | Vitamin E (mg) | Included | Vitamin |
| 21 | Folic acid (mcg) | Included | Vitamin |
| 22 | Iron (mg) | Included | Mineral |
| 23 | Magnesium (mg) | Included | Mineral |
| 24 | Zinc (mg) | Included | Mineral |
| 25 | Selenium (mcg) | Included | Mineral |
| 26 | Alcohol (g) | Included | Other |
| 27 | Caffeine (mg) | Included | Other |
| 28 | Tea (g) | Included | Other |
| 29 | Trans fat (g) | Unavailable | Fatty acid |
| 30 | Beta-carotene (mcg) | Unavailable | Vitamin |
| 31 | Anthocyanidins (mg) | Unavailable | Flavonoid |
| 32 | Flavan-3-ol (mg) | Unavailable | Flavonoid |
| 33 | Flavones (mg) | Unavailable | Flavonoid |
| 34 | Flavonols (mg) | Unavailable | Flavonoid |
| 35 | Flavonones (mg) | Unavailable | Flavonoid |
| 36 | Isoflavones (mg) | Unavailable | Flavonoid |
| 37 | Garlic (g) | Unavailable | Spice |
| 38 | Ginger (g) | Unavailable | Spice |
| 39 | Onion (g) | Unavailable | Spice |
| 40 | Saffron (g) | Unavailable | Spice |
| 41 | Turmeric (g) | Unavailable | Spice |
| 42 | Pepper (g) | Unavailable | Spice |
| 43 | Thyme/Oregano (g) | Unavailable | Spice |
| 44 | Rosemary (g) | Unavailable | Spice |
| 45 | Eugenol (mg) | Unavailable | Other |

Note: Of the 45 parameters in the original Shivappa protocol, 28 were available from our FFQ. The 17 unavailable parameters were primarily flavonoid subclasses and specific spices. This partial coverage is consistent with prior DII studies and may result in a narrower score range. MUFA, monounsaturated fatty acid; PUFA, polyunsaturated fatty acid; RE, retinol equivalents.

Supplementary Table S3. GMM fit comparison across 1-5 latent classes

| **Classes** | **Log-likelihood** | **AIC** | **BIC** | **SABIC** | **Entropy** | **Smallest class n (%)** | **Converged** |
| --- | --- | --- | --- | --- | --- | --- | --- |
| 1 | -3412.5 | 6833.0 | 6848.3 | 6835.4 | — | 324 (100%) | Yes |
| 2 | -3298.7 | 6611.4 | 6634.3 | 6614.8 | 0.78 | 89 (27.5%) | Yes |
| 3 | -3245.2 | 6510.4 | 6541.0 | 6514.8 | 0.74 | 65 (20.1%) | Yes |
| 4 | -3231.8 | 6489.6 | 6527.8 | 6495.0 | 0.71 | 28 (8.6%) | Yes |
| 5 | -3225.1 | 6482.2 | 6528.1 | 6488.6 | 0.68 | 14 (4.3%) | No |

Note: The 3-class model was selected as the optimal solution. Although the 4-class model showed marginally lower AIC, the 3-class solution offered better entropy, adequate smallest class size, and superior clinical interpretability. The 5-class model did not converge. AIC, Akaike Information Criterion; BIC, Bayesian Information Criterion; SABIC, Sample-Size Adjusted BIC. With only three time points, only linear slope and intercept could be estimated; nonlinear trajectories or random slope variance were not identifiable.

Supplementary Table 4a. LPA fit indices (original 6-variable specification)

| **Profiles** | **AIC** | **BIC** | **Entropy** | **BLRT P** | **Smallest class n (%)** |
| --- | --- | --- | --- | --- | --- |
| 2 | 5124.3 | 5178.6 | 0.82 | <0.001 | 98 (30.2%) |
| 3 | 4987.1 | 5064.2 | 0.76 | <0.001 | 54 (16.7%) |
| 4 | 4932.8 | 5032.7 | 0.73 | 0.012 | 22 (6.8%) |

Supplementary Table S4b. LPA 3-profile solution: cross-tabulation with GMM trajectory groups

| **LPA Profile** | **Rapid Recovery** | **Delayed Recovery** | **Chronic Persistent** | **Total** |
| --- | --- | --- | --- | --- |
| Profile 1 (Low-risk) | 42 | 58 | 8 | 108 |
| Profile 2 (Moderate) | 19 | 98 | 45 | 162 |
| Profile 3 (High-risk) | 4 | 26 | 24 | 54 |

Note: The 3-profile solution was selected based on overall fit and interpretability. Because the same variables were used for profiling and subsequent comparison, these results are descriptive. BLRT, Bootstrap Likelihood Ratio Test.

Supplementary Table S5. Restricted cubic spline Wald tests for DII and poor recovery

| **Test** | **Chi-square** | **df** | **P value** |
| --- | --- | --- | --- |
| Overall association | 7.9999 | 2 | 0.018 |
| Nonlinear component | 0.9366 | 1 | 0.333 |

Note: Model adjusted for age, sex, BMI, EJL, and baseline ODI. Three knots were placed at the 10th, 50th, and 90th percentiles of DII. The overall association was significant, but the nonlinear component was not, indicating that the data support an approximately linear adverse association rather than a J-shaped threshold effect.

Supplementary Table 6a. SEM global fit indices

| **Index** | **Value** | **Conventional threshold** |
| --- | --- | --- |
| CFI | 0.865 | > 0.90 preferred |
| RMSEA | 0.194 | < 0.08 preferred |
| SRMR | 0.060 | < 0.08 preferred |
| Chi-square (df) | 34.2 (4) | — |
| Chi-square P | < 0.001 | — |

Note: Only SRMR met the conventional threshold. CFI and RMSEA did not meet standard benchmarks, indicating suboptimal global fit. Results should therefore be interpreted cautiously as exploratory.

Supplementary Table 6b. Standardized structural path coefficients from SEM

| **Path** | **Std. Estimate** | **SE** | **z** | **P** | **Interpretation** |
| --- | --- | --- | --- | --- | --- |
| DII → IL-6 | 0.685 | 0.039 | 17.56 | < 0.001 | Strong positive |
| EJL → Sleep score | 0.279 | 0.052 | 5.37 | < 0.001 | Moderate positive |
| IL-6 → Pain VAS | 0.138 | 0.074 | 1.86 | 0.062 | Marginal, not significant |
| Pain VAS → Baseline ODI | 0.576 | 0.044 | 13.09 | < 0.001 | Strong positive |
| Sleep score → Baseline ODI | 0.033 | 0.053 | 0.63 | 0.530 | Not significant |
| IL-6 → Baseline ODI | −0.111 | 0.049 | −2.24 | 0.025 | Small negative |

Note: Estimation by maximum likelihood. The strong DII → IL-6 path and the strong Pain VAS → ODI path were the most robust findings. The IL-6 → ODI path was small, negative, and statistically significant, which is counterintuitive and may reflect residual confounding or model misspecification. The Sleep → ODI path was not significant.

Supplementary Table S7a. Univariable logistic regression for poor recovery (Chronic Persistent vs. others)

| **Variable** | **OR** | **95% CI** | **P value** |
| --- | --- | --- | --- |
| Age | 1.005 | 0.977 – 1.034 | 0.730 |
| Sex (Male vs Female) | 0.952 | 0.558 – 1.614 | 0.857 |
| BMI | 0.968 | 0.895 – 1.046 | 0.407 |
| Smoking (Yes vs No) | 0.960 | 0.510 – 1.756 | 0.899 |
| Drinking (Yes vs No) | 0.912 | 0.504 – 1.614 | 0.757 |
| Hypertension (Yes vs No) | 1.395 | 0.810 – 2.425 | 0.232 |
| Diabetes (Yes vs No) | 0.735 | 0.435 – 1.237 | 0.247 |
| HbA1c | 0.870 | 0.745 – 1.010 | 0.072 |
| DII | 1.186 | 1.001 – 1.411 | 0.049 |
| EJL | 0.870 | 0.543 – 1.377 | 0.554 |
| IL-6 | 1.254 | 0.990 – 1.598 | 0.063 |
| CRP | 1.310 | 0.895 – 1.935 | 0.168 |
| Sleep score | 1.282 | 1.178 – 1.404 | < 0.001 |
| Pain VAS | 3.218 | 2.505 – 4.275 | < 0.001 |
| Baseline ODI | 1.175 | 1.122 – 1.237 | < 0.001 |

Supplementary Table S7b. Multivariable logistic regression for poor recovery

| **Variable** | **OR** | **95% CI** | **P value** |
| --- | --- | --- | --- |
| DII | 1.256 | 0.811 – 1.993 | 0.317 |
| EJL | 0.648 | 0.273 – 1.457 | 0.307 |
| IL-6 | 0.929 | 0.529 – 1.600 | 0.793 |
| Sleep score | 1.223 | 1.050 – 1.442 | 0.012 |
| Pain VAS | 3.456 | 2.478 – 5.197 | < 0.001 |
| Baseline ODI | 1.153 | 1.072 – 1.254 | < 0.001 |

Note: Multivariable model adjusted for all variables listed. Only sleep score, pain VAS, and baseline ODI retained independent significance. DII, EJL, and IL-6 were not independently significant after mutual adjustment, suggesting that their prognostic relevance may operate through shared variance with symptom-level variables.

Supplementary Table 8. NHANES survey design and weighting specification

| **Element** | **Specification** |
| --- | --- |
| Cycles used | 2009-2010, 2015-2016, 2017-2018 |
| Weight variable | WTDRD1 / 3 (Day 1 dietary sample weight divided by number of cycles) |
| Stratification variable | SDMVSTRA |
| PSU variable | SDMVPSU |
| R package | survey (svydesign, svyglm) |
| Outcome definition | Self-reported low back pain in the past 3 months (variable BPQ060) |
| DII calculation | Based on Day 1 24-hour dietary recall; energy-adjusted per Shivappa protocol |
| Covariates | Age, sex, race/ethnicity, BMI, education, PIR, smoking, physical activity, total energy intake, EJL |

Note: NHANES uses a complex multi-stage probability sampling design. Appropriate survey weights must be applied to produce nationally representative estimates. The choice of WTDRD1 reflects the use of Day 1 dietary recall data for DII calculation.

Supplementary Table 9. ODI descriptive statistics by recovery trajectory group and time point

| **Time point** | **Rapid Recovery (N=65)** | **Delayed Recovery (N=182)** | **Chronic Persistent (N=77)** | **Overall (N=324)** |
| --- | --- | --- | --- | --- |
| Baseline ODI, mean (SD) | 52.82 (8.57) | 62.00 (6.97) | 69.71 (7.57) | 62.00 (9.30) |
| 3-month ODI, mean (SD) | 22.15 (8.42) | 38.50 (9.13) | 58.34 (10.25) | 39.12 (15.47) |
| 6-month ODI, mean (SD) | 8.92 (5.63) | 20.44 (7.86) | 52.18 (11.30) | 25.67 (17.82) |
| ODI change baseline→6m | −43.90 (9.21) | −41.56 (8.74) | −17.53 (10.88) | −36.33 (14.56) |

Note: ODI, Oswestry Disability Index (0-100, higher = worse disability). The Chronic Persistent group showed the smallest absolute ODI reduction over 6 months.

Supplementary Table S10. Baseline characteristics of NHANES participants by DII quartile

| **Characteristic** | **Overall**  N = 160,946,252^1^ | **Q1 (Anti-inflammatory)**  N = 44,721,817^1^ | **Q2**  N = 41,171,923^1^ | **Q3**  N = 39,685,365^1^ | **Q4 (Pro-inflammatory)**  N = 35,367,147^1^ | **p-value**^2^ |
| --- | --- | --- | --- | --- | --- | --- |
| **RIDAGEYR** | 48.45 (17.23) | 48.46 (16.27) | 48.04 (16.84) | 48.58 (17.75) | 48.76 (18.25) | 0.7 |
| **Gender** |  |  |  |  |  | <0.001 |
| Male | 5,586 (49.5%) | 1,803 (64.6%) | 1,510 (53.1%) | 1,260 (42.8%) | 1,013 (33.8%) |  |
| Female | 5,707 (50.5%) | 1,021 (35.4%) | 1,313 (46.9%) | 1,563 (57.2%) | 1,810 (66.2%) |  |
| **Race** |  |  |  |  |  | <0.001 |
| Mexican American | 1,900 (8.5%) | 512 (9.1%) | 490 (8.6%) | 471 (8.1%) | 427 (7.9%) |  |
| Other Hispanic | 1,242 (5.9%) | 294 (5.6%) | 335 (6.5%) | 286 (5.0%) | 327 (6.6%) |  |
| Non-Hispanic White | 4,676 (67.7%) | 1,226 (70.0%) | 1,185 (68.6%) | 1,183 (68.1%) | 1,082 (63.2%) |  |
| Non-Hispanic Black | 2,148 (9.9%) | 378 (6.1%) | 483 (8.6%) | 579 (11.1%) | 708 (14.8%) |  |
| Other/Multi | 1,327 (8.1%) | 414 (9.2%) | 330 (7.8%) | 304 (7.7%) | 279 (7.5%) |  |
| **BMXBMI** | 28.57 (4.85) | 28.23 (4.62) | 28.55 (4.82) | 28.74 (4.99) | 28.83 (4.99) | 0.053 |
| **BMXWAIST** | 98.78 (13.35) | 98.53 (12.99) | 98.92 (13.34) | 99.01 (13.70) | 98.70 (13.43) | 0.9 |
| **Smoking** |  |  |  |  |  | 0.001 |
| Non-Smoker | 6,454 (57.1%) | 1,655 (58.8%) | 1,635 (59.0%) | 1,615 (57.2%) | 1,549 (52.5%) |  |
| Smoker | 4,839 (42.9%) | 1,169 (41.2%) | 1,188 (41.0%) | 1,208 (42.8%) | 1,274 (47.5%) |  |
| **Alcohol** |  |  |  |  |  | <0.001 |
| Drinker | 8,381 (79.6%) | 2,260 (84.7%) | 2,165 (81.2%) | 2,047 (78.8%) | 1,909 (72.1%) |  |
| Non-Drinker | 2,912 (20.4%) | 564 (15.3%) | 658 (18.8%) | 776 (21.2%) | 914 (27.9%) |  |
| Diet_Jetlag | 1.25 (1.33) | 1.13 (1.16) | 1.18 (1.22) | 1.28 (1.39) | 1.45 (1.55) | <0.001 |
| LBP_Status | 1,641 (13.4%) | 323 (9.9%) | 405 (13.2%) | 435 (14.8%) | 478 (16.4%) | <0.001 |
| Hypertension | 4,020 (31.2%) | 916 (28.3%) | 988 (31.1%) | 1,046 (33.4%) | 1,070 (32.5%) | 0.036 |
| LBXHSCRP | 2.42 (5.10) | 1.76 (3.42) | 2.20 (4.67) | 2.70 (4.73) | 3.22 (7.20) | <0.001 |
| DII | 0.97 (2.28) | -1.96 (1.03) | 0.46 (0.56) | 2.21 (0.44) | 3.89 (0.61) | <0.001 |
| DR1TKCAL | 2,156.50 (952.58) | 2,865.53 (1,067.56) | 2,337.96 (737.14) | 1,867.33 (584.96) | 1,373.17 (539.35) | <0.001 |
| DR1TFIBE | 17.16 (10.41) | 27.42 (11.28) | 17.92 (6.93) | 12.42 (5.00) | 8.65 (4.12) | <0.001 |
| DR1TPROT | 83.00 (41.77) | 114.83 (46.07) | 90.10 (33.44) | 70.31 (26.39) | 48.73 (21.85) | <0.001 |
| DR1TTFAT | 85.32 (46.47) | 114.15 (55.97) | 93.02 (39.17) | 73.66 (30.51) | 52.99 (26.82) | <0.001 |
| ^1^Mean (SD); n (unweighted) (%) | | | | | | |
| ^2^Design-based KruskalWallis test; Pearson's X^2: Rao & Scott adjustment | | | | | | |
